# P035 Not all that glistens is arteritis

**DOI:** 10.1093/rap/rkag113.065

**Published:** 2026-11-03

**Authors:** Meena Naja, George Nowell, Lisa Waters, Ellen Bruce

**Affiliations:** Manchester University NHS Foundation Trust, Manchester, United Kingdom; Manchester University NHS Foundation Trust, Manchester, United Kingdom; Manchester University NHS Foundation Trust, Manchester, United Kingdom; Manchester University NHS Foundation Trust, Manchester, United Kingdom

## Abstract

**Introduction:**

Giant cell arteritis (GCA) is the commonest vasculitis worldwide. It frequently involves the temporal arteries (TA) and can be complicated by irreversible visual loss. Suspected GCA cases comprise a significant proportion of urgent rheumatology referrals. There is no single diagnostic test; clinical assessment includes history, examination, the Southend GCA probability score (GCAPS), blood tests, ultrasound and biopsy. The diagnosis is ultimately clinical. Many conditions can closely mimic GCA which can lead to diagnostic uncertainty and excessive steroid burden. Here we describe a case of Monckeberg medial calcific sclerosis (MMCS), a rare GCA mimic.

**Case description:**

A 90-year-old Greek woman presented to eye casualty. She had an extensive medical history including chronic kidney disease (CKD), atrial fibrillation, aortic stenosis, heart failure, osteoporosis, frailty, recurrent falls, and cataracts.

She initially presented with acute painless blurred vision in the right eye, confirmed as central retinal artery occlusion (CRAO) by ophthalmology. She had been experiencing non-specific jaw and shoulder pain for several months prior. No initial treatment was given by ophthalmology and she was discharged with a plan for follow-up in the stroke clinic.

She re-presented three weeks later with blurred vision in the left eye. She was noted to have bilateral thickened TAs, with tenderness and weak pulsation in the right TA. Blood results showed modest inflammatory markers (CRP 10, ESR 49) and mild normocytic anaemia. Initial GCAPS was 18 (high risk) based on: age, time of onset, ischaemic symptoms, temporal artery examination findings, and CRAO. She was treated by ophthalmology with intravenous methylprednisolone followed by high-dose oral prednisolone. There was no symptomatic improvement with steroid treatment.

Initial rheumatology opinion was of high pre-test probability of GCA. Temporal artery ultrasound showed no halo sign, the frontal and parietal branches of the TAs were calcified but compressible. A TA biopsy (TAB) showed extensive disruption of internal elastic lamina with irregular calcific deposits without inflammatory infiltrate, consistent with MMCS.

Steroids were rapidly tapered without deterioration of symptoms, and vascular risk factors were addressed. Other than worsening fatigue, the patient did not experience any acute complications of high-dose steroid treatment despite her multiple risk factors for significant steroid toxicity.

**Discussion:**

Monckeberg medial calcific sclerosis (MMCS) is a chronic non-inflammatory vascular condition characterised by calcification of the tunica media of arteries. The prevalence is estimated at 1.6—10%. It is associated with advancing age, male sex, diabetes and CKD. Unlike atherosclerosis, there is no involvement of the tunica intima. The condition is often an incidental finding on plain film X-ray of the lower limbs (characteristic ‘railroad track’ pattern of calcification). An association has been shown between MMCS and increased risk of critical lower limb ischaemia, future cardiovascular events, and all-cause mortality, potentially due to increased arterial stiffness.

There have been several case reports published to date describing cases of MMCS presenting as a GCA mimic. A literature review in 2025 identified eleven cases that presented with classical GCA features, including headache, jaw claudication and visual disturbance, including one previous case with CRAO. All of these cases, like ours, were treated empirically with high-dose glucocorticoids prior to the diagnosis of MMCS on TAB.

This case illustrates some of the diagnostic challenges posed by GCA mimics. Clinical features traditionally associated with high probability of GCA (such as advanced age, ischaemic visual symptoms and abnormal temporal artery examination) may also be present in MMCS and other mimics.

This is now the second described case where MMCS presented with CRAO and temporal artery thickening. The visual disturbance and temporal artery thickening on clinical examination were the major contributors to the decision to continue steroid treatment in this patient despite an atypical history and negative ultrasound. This case highlights the value of TAB in similar scenarios of diagnostic uncertainty.

It remains unclear whether the MMCS directly caused the CRAO, or whether these are two distinct pathologies in a patient with multiple vascular risk factors.

**Key learning points:**

• This case illustrates that visual disturbance with temporal artery thickening, tenderness, and reduced pulsation is not pathognomonic of GCA. GCA is a clinical diagnosis that is supported, but not defined, by investigations. Discordance between clinical features, imaging and treatment response should prompt reassessment rather than diagnostic anchoring. Temporal artery biopsy remains a valuable investigation in cases of diagnostic uncertainty.

• It is the role of the rheumatologist to make a reasoned clinical judgement in situations of uncertainty. Where the balance of evidence shifts away from GCA, the rheumatologist often must act with courage to withdraw potentially harmful corticosteroid therapy. This requires careful synthesis of clinical, laboratory and histological findings, and a willingness to revisit an initial working diagnosis in the best interests of the patient, despite the perceived risks on either side. The risk of corticosteroid-related harm is amplified in the cohort of patients who are referred with suspected GCA, as these will be older people who are more likely to have pre-existing comorbidities.

• From the Case-based Conference, we would like to gain the following learning objectives:

1. Managing uncertainty in GCA referrals, in particular balancing the risks of corticosteroid-related harm against the potential consequences of undertreated disease.

2. Increasing awareness of common, and less common, GCA mimics. Understanding that some mimics can present with classical GCA features (jaw pain, TA thickening, visual disturbance), reinforcing the need for a holistic clinical assessment.

3. Pros and cons of GCAPS: to be used as a structured tool while understanding its limitations.

